# PBPK Modeling on Organs-on-Chips: An Overview of Recent Advancements

**DOI:** 10.3389/fbioe.2022.900481

**Published:** 2022-04-14

**Authors:** Yi Yang, Yin Chen, Liang Wang, Shihui Xu, Guoqing Fang, Xilin Guo, Zaozao Chen, Zhongze Gu

**Affiliations:** ^1^ State Key Laboratory of Bioelectronics, School of Biological Science & Medical Engineering, Southeast University, Nanjing, China; ^2^ Jiangsu Provincial Center for Disease Control and Prevention, Key Laboratory of Enteric Pathogenic Microbiology, Ministry Health, Institute of Pathogenic Microbiology Health, Nanjing, China; ^3^ Institute of Medical Devices (Suzhou), Southeast University, Suzhou, China

**Keywords:** PBPK modeling, Organ-on-a-chip, drug development, pharmacokinetic, preclinical trails

## Abstract

Organ-on-a-chip (OoC) is a new and promising technology, which aims to improve the efficiency of drug development and realize personalized medicine by simulating *in vivo* environment *in vitro*. Physiologically based pharmacokinetic (PBPK) modeling is believed to have the advantage of better reflecting the absorption, distribution, metabolism and excretion process of drugs *in vivo* than traditional compartmental or non-compartmental pharmacokinetic models. The combination of PBPK modeling and organ-on-a-chip is believed to provide a strong new tool for new drug development and have the potential to replace animal testing. This article provides the recent development of organ-on-a-chip technology and PBPK modeling including model construction, parameter estimation and validation strategies. Application of PBPK modeling on Organ-on-a-Chip (OoC) has been emphasized, and considerable progress has been made. PBPK modeling on OoC would become an essential part of new drug development, personalized medicine and other fields.

## 1 Introduction

To predict the pharmacokinetic (PK) behaviors of drug absorption, distribution, metabolism and excretion (ADME) in a quantitative manner is of great importance in new drug development. PK modeling uses mathematical principles and methods to study the dynamics behaviors of drugs *in vivo* like ADME. Before conducting clinical trials of the drug, it is necessary to establish an effective PK model to analyze and predict the kinetics of multi-organ interactions, the concentration curve of drug and its metabolites over time to determine the appropriate dose of the drug. Pharmacodynamics (PD) studies the effect of a drug *in vivo*, including efficacy (drug action on tissues or organs), toxicity (side effects), and drug-drug interactions (effects of drug combinations). The combined PK-PD model can be used to predict and analyze the physiological effect of a drug on organs at a given dose. Physiologically based pharmacokinetic (PBPK) model studies the drug pharmacokinetics by viewing human organs as separate compartments and integrating them into a system according to physiological and anatomical knowledge. Compared with the traditional compartmental pharmacokinetic model, the compartments and model parameters of PBPK model have physiological meaning. Thus PBPK model is believed to more accurately reflect the ADME process of the drug *in vivo*.

The failure of preclinical cell culture and animal models to predict the efficacy and safety of drugs *in vivo* causes huge amounts waste of time, resources and money each year (Edington et al., 2018). Besides animal experiments are also under increasing ethical pressure. Since proposed in 1959, the 3R principle (i.e., replacement, reduction, refinement) has been incorporated into legislation when conducting animal experiments for scientific purposes [e.g., European Union’s 2010/63/EU Directive (D´ıaz et al., 2021)]. Under the guidance of the 3R principle, in 2019, the US Environmental Protection Agency proposed to phase out any experiments on mammals by 2035 (U.S. EPA Holds Inaugural Conference on Reducing Animal Testing for Chemical Safety, 2019). Some scholars recommended using new methods (Fernandes and Pedroso, 2017) to replace animal experiments. These methods include *in vitro* tests (tissues and cells); non-invasive clinical research on human volunteers; the use of species (e.g., shrimp and daphnia larvae) that are not listed as protected animals; computer simulation, etc. The above events have led to a series of emerging technologies that capture complex human physiology *in vitro* outside the human body, including the micro-physiological system (MPS), also known as the organs-on-a-chip (OoC). OoC technology is considered to have the potential to improve the efficiency of drug development and replace animal experiments (Edington et al., 2018; Heringa et al., 2020; Chen et al., 2021; Lee et al., 2021; Yang et al., 2021; Zhang et al., 2021). The combination of OoC technology and PK-PD model provides a new tool for drug development.

## 2 Organ-On-a-Chip Overview

### 2.1 Organ-on-a-Chip

**FIGURE 1:**
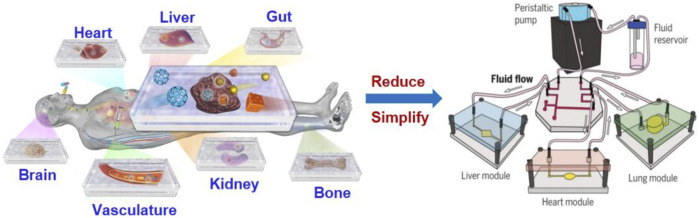
Illustration of organ-on-a-chip technologies to mimic human organs structure and function (Park et al., 2019).

Organ-on-a-chip (OoC) is a kind of microfluidic cell culture equipment, which reproduces the physiological and pathological characteristics of organs or tissues by reconstructing the structure and function *in vitro* (Figure 1). The design and fabrication of an organ-on-a-chip system involves a series of technologies, including microfluidic, material and cell biology which will be introduced in the following subsections.

#### 2.1.1. Microfluidic

Microfluidics refers to the technology for precisely manipulating microscale fluids in tiny structures (usually on the sub-micron scale), also known as lab-on-a-chip. The combination of microfluidic technology and cell culture technology has created OoC technology that is believed to be able to capture complex human physiology *in vitro*. Microfluidics can control the cell culture conditions on the microscopic level (Wikswo et al., 2013b; Jiang et al., 2016), and the geometric design of the organ chip can further control the biochemical phenomena related to the concentration gradient, such as the formation of blood vessels (Zhang et al., 2016). Linda (Edington et al., 2018) reported a programmable microfluidic system that controls the rate of fluid in the organ chip by changing the frequency of compressed air and partial vacuum delivered by the manifold.

#### 2.1.2 Material

Most of the current OoC cell culture devices are made of polydimethylsiloxane (PDMS). However, the adsorption of small hydrophobic molecules by PDMS makes it challenging to accurately predict the results of drug experiments. Ingber (Grant et al., 2021) reported their work on predicting the drug concentration of organ chips through simulation and experimental methods with the molecular adsorption process taken into consideration. In addition, some materials such as polysulfone (PSF) plastic (Edington et al., 2018) are considered to have the potential to replace PDMS for their lower absorption ability of hydrophobic molecules compared with PDMS.

Thin and flexible biopolymer membranes also play an important role in making culture devices for OoC. Such structures can be used to simulate the specific biological characteristics of organs, such as mechanical stretching to simulate the breathing process. Polyurethane membrane (Gabriel et al., 2017) has been reported as a material for fabricating this type of biopolymer membrane.

#### 2.1.3 3D Cell Culture

A specific function of an organ may rely on its specific cell structures. A series of 3D structures (Lee et al., 2019; Oso´rio et al., 2021; Shim et al., 2017) have been proposed to better mimic human physiology. 3D bioprinting is currently one of the most promising technologies for shaping cell structure, and has been successfully applied in the production of artificial organ chips (Ewald et al., 2021).

### 2.2 Recent Reported Progress on Organ-on-a-Chip Technology

#### 2.2.1 Heart-on-a-Chip

The heart is responsible for providing sufficient blood to other organs and tissues in the human body to supply oxygen and nutrients and take away the metabolic waste in the organs. Zhang (Zhang et al., 2016) proposed a novel hybrid strategy based on 3D bioprinting to fabricate endothelialized myocardium. By using composite bio-ink, the endothelial cells are directly bioprinted in the microfiber hydrogel scaffold to form a layer of confluent endothelium. An aligned myocardium capable of spontaneous and synchronous contraction can be made by seeding cardiomyocytes into the 3D endothelial bed.

#### 2.2.2 Intestine-on-a-Chip

The intestine is an important digestive organ of the human body. Oral drugs must pass through the small intestine to enter the blood. Therefore, research on intestinal chips is essential for preclinical drug experiments using OoC technology. Zhang (Zhang et al., 2016) combined intestinal tissue engineering and OoC technology to establish an *in vitro* biological model of the human duodenum. The intestinal epithelial cells cultured on the chip come from endoscopic biopsy or organ resection. This chip represents the closest model to the living duodenum and captures the key features of the small intestine.

#### 2.2.3 Liver-on-a-Chip

The liver system is the main site of drug metabolism. The liver is composed of a series of complex hepatic lobules (Mccuskey, 2008). Maintaining the long-term physiological function of liver cells is a challenging problem. With the purpose of improving the physiological model of hepatocytes *in vivo*, 3D hepatocyte culture technology is applied (An et al., 2015; Ma et al., 2018) to develop a platform that presented a long-term maintenance of liver-on-a-chip. Yum (Yum et al., 2014) developed a system to study how liver cells affect other cell types. A high-throughput assay was developed to assess the toxicity of hepatocyte drugs. Riahi (Riahi et al., 2016) fabricated a microfluidic electrochemical chip sensor to detect biomarkers produced in the process of liver metabolism. Liver-on-a-Chip has shown great application value in the field of clinical trial. Hou (Hou et al., 2020) developed an integrated biomimetic array chip (iBAC) including a Liver-on-a-Chip and a tumor-on-a-Chip as a screening tool of drug development. Xiao (Xiao et al., 2021) developed an iBAC for establishing extracellular matrix (ECM) based model as a case for systematical prediction of hepatotoxicity.

#### 2.2.4 Pancreas-on-a-Chip

Pancreas-on-a-chip technology is rapidly growing into a platform for complex *in vitro* modeling of pancreatic islet physiology. Advances in microfluidic design through the use of imaging compatible bio-materials and biosensor technology may provide a new future tool for predicting the outcome of islet transplantation. The progress of combining pancreatic islets with other types of tissues provides the possibility to study diabetes intervention in a minimally equivalent *in vitro* environment (Abadpour et al., 2020). With the purpose to investigate the cause of cystic fibrosis, A pancreas-on-a-chip incorporated in an *in vitro* co-culture system was proposed to deepened the understanding of pancreatic function (Shik Mun et al., 2019).

#### 2.2.5 Vascularized Organ-on-a-Chip

The blood vessel itself is a major organ in the human body, which carries nutrients, immune cells, signaling molecules and therapeutic drugs to all other organs. It also plays a key role in inducing and maintaining tissue characteristics and providing a tissue-specific environment (i.e., vascular niche) that supports the survival and function of stem cells through the expression of vascular secretion factors.

Vascularized OoC are currently a promising research direction in the field of organ chips. There are at least three reasons for the application of Vascularized OoC: 1) Replication of vascular secretion signals (Osaki et al., 2018). Growth factors secreted by vascular endothelial cells are considered to be essential in inducing organ regeneration, as well as the maintenance of homeostasis and metabolism; 2) Building of a larger-scale *in vitro* tissues (Mehling and Tay, 2014; Jung et al., 2015), beyond the volume limitation of traditional *in vitro* tissues that rely on diffusion for nutrient delivery and waste elimination; 3) Capture the complex human physiology *in vitro* (Ji et al., 2016; Chen et al., 2018). There are a large number of organ epithelial cells and vascular endothelial cells in the human body with of physiologically relevant tissue-tissue barriers between vascular endothelial cells and parenchymal cells of various organs. These barriers can significantly affect the ADME process *in vivo*.

#### 2.2.6 The Fabrication of Vascularized Organ-on-a-Chip

Capillary-like structures in tissue-engineered organs are generally produced through self-assembled, pre-patterned, and 3D-bioprinted (Rollas, 2008). The self-assembled vascularized OoC platform reproduces the formation of new blood vessels *in vitro*, the pre-patterned and 3D-bioprinted vascularized OoC can form a fully perfusable vascular system with predefined dimensions and control configurations, but the size of the blood vessels is largely limited by the resolution and size of the mold or nozzle.

#### 2.2.7 Advantages of Vascularized Organ-on-a-Chip

There are substantial epithelial cells of organs and endothelial cells of blood vessels in the human body, and physiologically related tissue-tissue barriers exist between the vascular endothelium and the parenchymal cells of each organ. These barriers can significantly affect the ADME response. This is essential for simulating PK in a clinically relevant way. Vascularized OoC will be helpful in simulating the complex mechanism of multi-organ cooperation *in vivo*.

### 2.3 Multiple Organs-on-Chips (Human-on-a-Chip)

Since researchers have made great progress (Grosberg et al., 2011; Mehling and Tay, 2014) on Individual OoC technologies in the last decade, it is still difficult to put these technologies into the use of real drug experiment before multiple organs can be integrated into a single system correctly. Even though our understanding of a multiple coupled OoCs or human-on-a-chip (HoC) is very limited so far, some progress has been achieved by scaling and system biology for multiple OoCs as explored in several studies. An introduction to these studies will be provided.

John (Wikswo et al., 2013b) argues that “To replicate human physiology and drug response with interconnected human OoCs/HoCs, it is critical that each OoC/HoC has the correct relative size.” By observing a large number of plants and animals, scientists have discovered formulas that can express the relationship between parts and the whole organism, which is called allometric scaling. Allometric scaling formula has the form *M* = *AM* ^*B*^. *M*_*b*_ represents the mass of an organism and A, B represent coefficients related to Species. M here represents the mass of an organ. It is necessary to establish the relationship between human mass and organ mass before applying this formula into determining micro-organ relative size.

**FIGURE 2:**
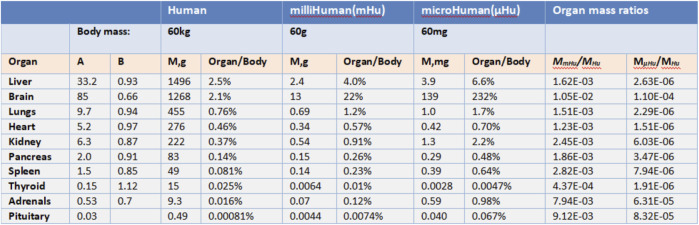
Allometric scaling coefficients and organ masse (Adapted from Wikswo et al., 2013b).

Allometric scaling has been extremely little used in the research of multiple OoCs while it has been studied for more than one century. Stahl (1965) puts it that allometric scaling can be applied to determining the human organs mass according to the human body mass using the statistics collected from primates (Alford et al., 2010). Figure 2 shows coefficients A and B obtained from primates. These coefficients inform us how the weight of different organs or tissues should be determined based on the allometric scaling law for a multiple-organs-on-chips (MOoCs).

However, there still exists considerably problems in the application of allometric scaling. First, allometric scaling may not give the appropriate organs mass estimation since the coefficients are derived from primates weighed over 10 g. If the mass of a human body is set as 60 mg, the mass of the brain will be 139 mg according to allometric scaling which is obvious not rational. In this situation, the mass of different organs on a chip must be modified by researchers to make sure they have correct relative size and all modifications are based upon the experience of researchers. Second, OoCs may be impossible to conduct drug experiments if its mass is decided by allometric scaling. As Grosberg (Grosberg et al., 2011) puts certain immune cells function at such a low density [3,000 leukocytes per ml of cerebral spinal fluid (CSF)] that the breadth of acquired immune response may not be replicable in a µBrain with a CSF volume around 1 µl that would contain about 3 leukocytes. It is obvious that a brain immune drug experiment cannot be conducted on the brain-on-a-chip since it has too little leukocytes while the mass of the brain is relatively large.

Since the application of allometric scaling in integrating OoCs still remains challenging. researchers attempt to find other solutions to integrate multiple organs into a single system. Grosberg has descripted it as “living histological ‘section’ of an adult human” (Grosberg et al., 2011). Constructing a single OoC which is relatively big and can reflect the property of a human organ, then put these different single OoC together.

On the condition that the volume for the media of a certain OoC is decided properly, Grosberg (Grosberg et al., 2011) argues that “It is necessary, however, to make the ‘section’ large enough and sufficiently realistic that the organ functions in a more physiologically realistic manner than a simple monolayer monoculture in a Petri dish or well plate. Building a functional ‘section’ from an individual human’s cells may have advantages over using real ones in that it may be possible to create ‘sections’ of an individual patient’s organs that are not readily available.”

While living histological “section” performs well in the experiment on a single OoC, it is complex to integrate two or more OoCs in a system. The size of each section must be modified carefully to achieve a system that can correctly recover the interaction between different organs.

Obviously, it is very difficult to simulate the human body system even from the mass level. The researchers also proposed a method (Alford et al., 2010; Grosberg et al., 2011) which is called functional scaling that does not approximate the human body in every aspect but focuses on the main indicators of each organ chip. For example, we only pay attention to the gas exchange ability of a lung (Gas exchange volume per unit time). This method provides a realistic way to integrate multiple OoCs into a coupled system. However, functional scaling may lead to an oversimplification of micro physiology system and increase the difficulty of interpreting experimental results.

The current common practice in establishing the application of human-on-a-chipis as follows: multiple organs on the chip are simultaneously cultured with cells from different organs and tissues. These cells are connected through channels to achieve the integration of multiple organs to establish a system (Palaninathan et al., 2018; Zhao et al., 2019). These methods can be divided into static, semi-static and flexible methods (Rogal et al., 2017). Static multiple organs are integrated into a single connected device. In a semi-static system, organs are connected by a fluid network through a tissue insert based on Transwell® (Rogal et al., 2017). In systems that use flexible methods, individual organ-specific platforms are interconnected using flexible micro-channels. Such a system facilitates the recreation of multiple organs (Rogal et al., 2017). Although the concept of multi-organ chips is still in its infancy, some breakthroughs have been made, including dual-organ (van Midwoud et al., 2010; Tsamandouras et al., 2017), three-organs (Maschmeyer et al., 2015; Skardal et al., 2017), four-organs (Maschmeyer et al., 2015; Oleaga et al., 2016) and ten-organs on-chip (Edington et al., 2018) design.

## 3 Physiologically Based Pharmacokinetic Modeling

### 3.1 Physiologically Based Pharmacokinetic Models

#### 3.1.1 The Development of PK/PD Model

**FIGURE 3:**
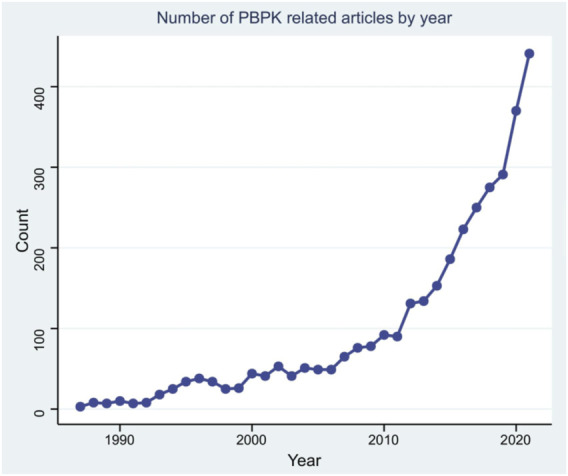
Number of PBPK related articles by year.

Although some explorations on ADME process of drugs *in vivo* have been done before 1937 (Widmark and Tandberg, 1924; Paalzow, 1995), the establishment of modern pharmacokinetics is generally attributed to Torsten (Paalzow, 1995) in 1937 for his proposition of two-compartment model. In 1960, Brodie (Brodie et al., 1960) introduced a calculation method of pharmacokinetic model parameter for the first time when studying the drugs entering the cerebrospinal fluid. In 1978, Yamaoka (Yamaoka et al., 1978) applied the statistical moment method to pharmacokinetics research, which was later known as the non-compartmental model of pharmacokinetics. Since then, pharmacokinetics has gone through a period of rapid development, during which the PBPK modeling has been established. To count the relevant data, a PubMed search was conducted using the search terms “PK/PD,” “pharmacokinetics” and “pharmacodynamics” within the abstract or title of the manuscript. The results are shown in the Figure 3. As our knowledge of human physiology advances, the PBPK model has become more complex and closer to the actual situation of human body. More commercial PBPK modeling software such as Gastroplus® and Simcpy® has emerged to study the ADME behaviors of the drugs. In the future, the PBPK model will be applied to different fields including food safety, and will be more precise and more physiologically sound.

#### 3.1.2 Physiologically Based Pharmacokinetic Models

**FIGURE 4:**
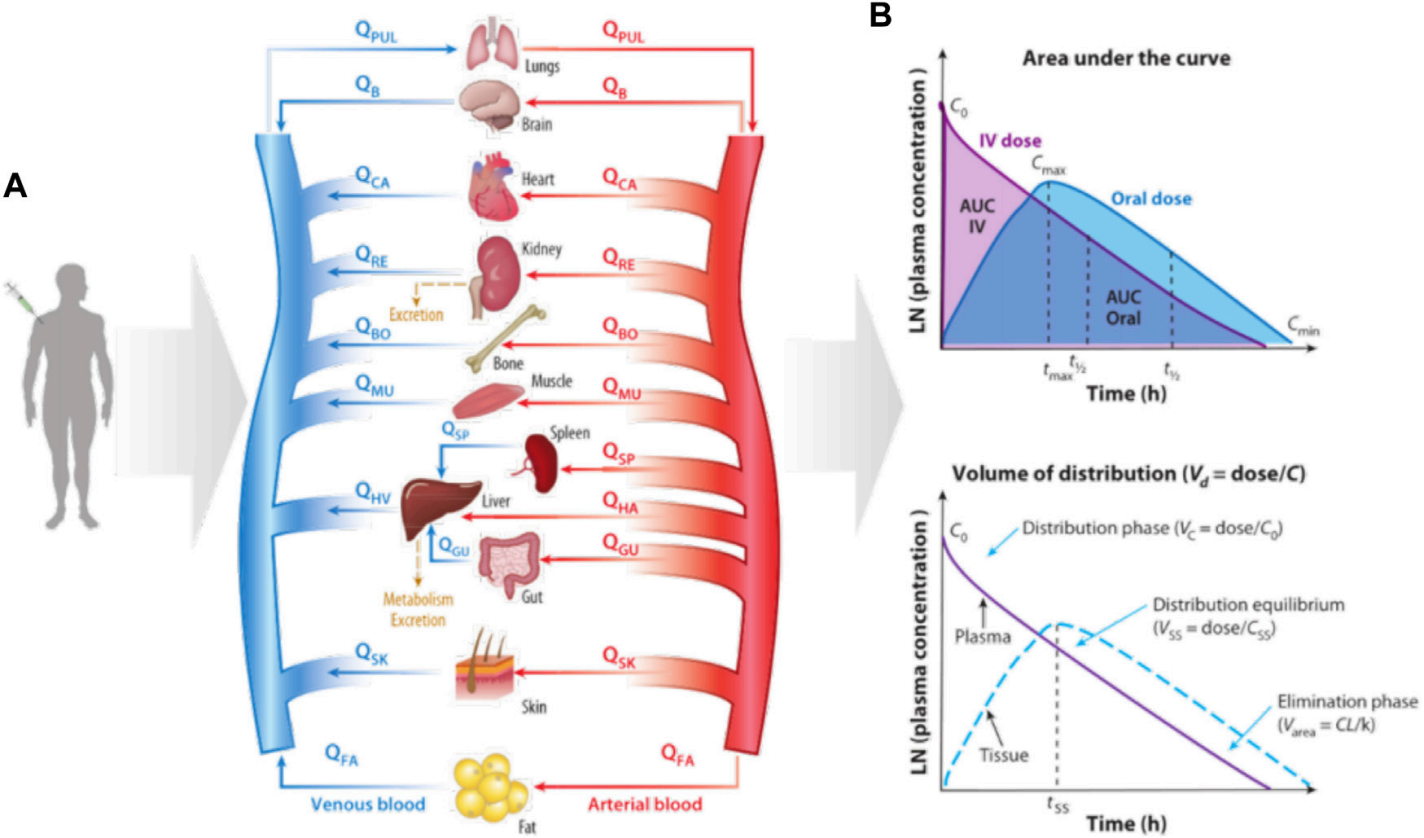
Diagram of how organ size, composition, and blood flows (Q) are used to construct a physiologically based pharmacokinetic (PBPK) model of the whole body and predict the kinetics of drug exposure (Prantil-Baun et al., 2018). **(A)** How a PBPK model can be built based on physiology knowledge; **(B)** The calculation of key PK parameters.

Physiologically based PK (PBPK) models (Rollas, 2008; Rowland et al., 2011; Sager et al., 2015) have been developed to simulate drugs behavior in complex human body. The PBPK model represents the human body as a network connecting various organs through arterial and venous blood flow (Figure 4) (Prantil-Baun et al., 2018). The red arrow indicates the blood flow direction of the artery and the blue arrow indicates the blood flow direction of the vein. Figure 4B gives how some key PK parameters can be calculated from drug exposure curves. The graph on the top shows the area under the curve (AUC), which reflects the time dependent exposure in the body to a drug over time, and shows how drug levels vary with administration, as shown by comparing oral and intravenous (IV) dosing. From these curves, one can identify the maximum concentration after initial administration of an IV drug (C0), maximum concentration for orally administered drug (Cmax), and minimum concentration at the end of the elimination phase (Cmin), as well as the time it takes for the plasma concentration to decrease by 50% (t1/2) and the corresponding time (tmax) to reach Cmax after oral administration of the drug. The bottom graph shows the determination of volume of distribution (Vd). Vc represents the volume in which the drug is distributed immediately after IV administration when the drug is limited to the blood and, thus, highly perfused organs. The steady-state volume of distribution (Vss) is achieved when the drug enters tissues. The parameter Varea describes the elimination phase of the drug, whether it is metabolized or cleared from the system, typically by the liver and kidney, where CL represents clearance and k is the elimination rate constant, which describes the rate at which a drug is removed from the system. Some key PK parameters, such as maximum concentration (Cmax), maximum time to reach Cmax (tmax), area under the curve (AUC), bioavailability (F), volume of distribution (Vd), clearance (CL), elimination rate constant (k), and half-life (t1/2) are described briefly here (Benet, 1984; Urso et al., 2002; Rowland and Tozer, 2005; Aungst, 2017).

### 3.2 The Establishment of Physiologically Based Pharmacokinetic Models

PBPK models can be divided into two types: the full model and the minimal model. The full PBPK models mean that all distribution organs and tissues are represented as independent perfused chambers, and the minimum PBPK models lump organs with similar kinetics. The full PBPK models often fit the experimental data better than the minimum model, but too many parameters may lead to overfitting. An advantage of the full PBPK modeling is that it can simulate the exposure of drugs or their metabolites in specific tissues that are not available for clinical sampling. The pharmacological or toxicological effects of some drugs are driven by the concentration in the tissue, which is particularly important. The complete PBPK model is usually used to systematically predict the distribution kinetics to simulate the plasma concentration-time distribution.

**FIGURE 5:**
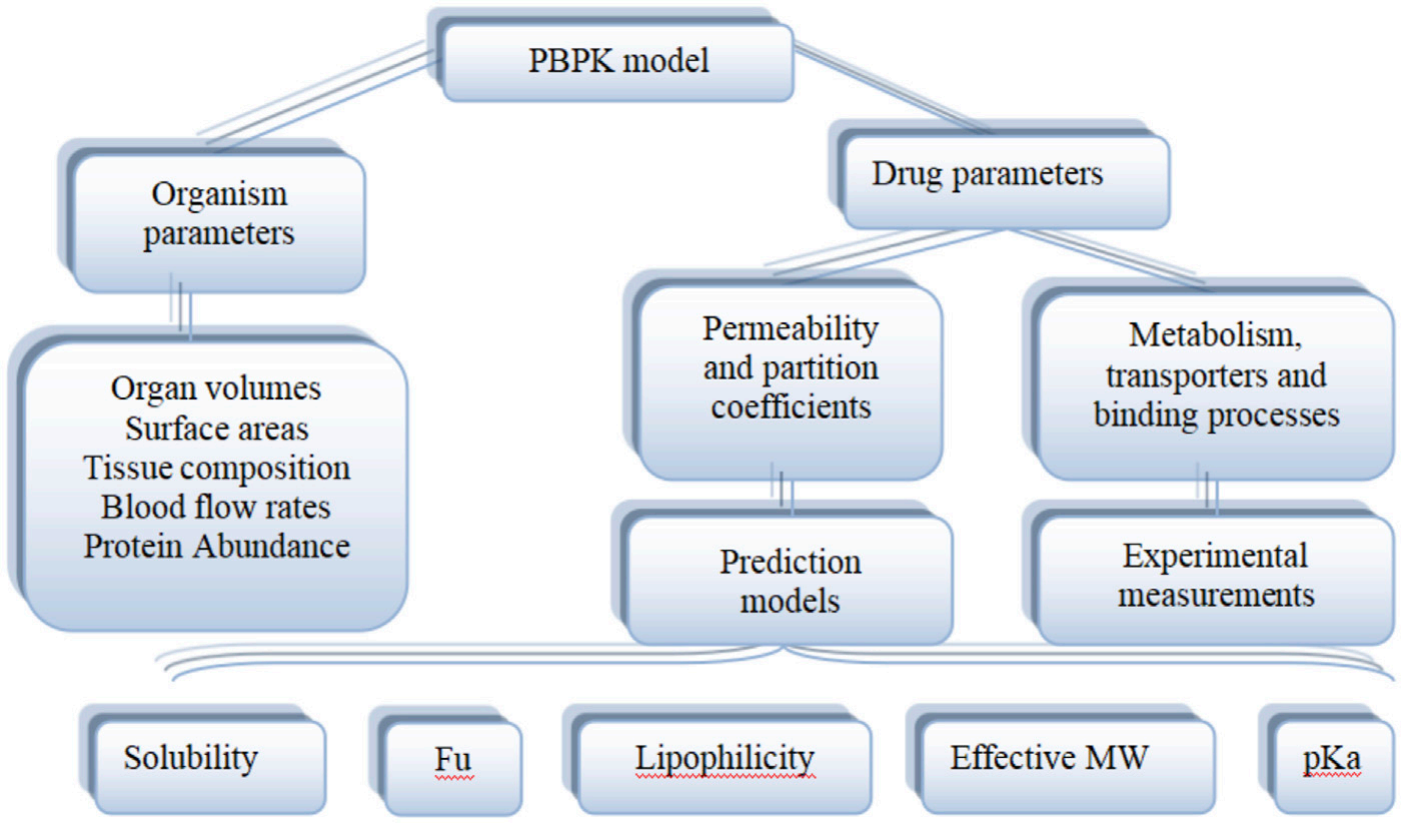
Physiological and drug parameters in PBPK models (Adapted from Kuepfer et al., 2016).

As shown in Figure 5, the PBPK model consists of system-specific and drug-specific parameters. System- specific parameters include blood flow, organ volume, expression of enzymes and transporters, and plasma protein concentration. Drug-specific parameters include internal clearance, volume of distribution, solubility and physical and chemical parameters, tissue distribution, plasma protein binding affinity and membrane permeability. Drug-related parameters are independent of system-specific parameters, so the *in vitro* bottom-up method can be used to mechanically extrapolate human body dynamics parameters. Drug dependent parameters are independent of system parameters, allowing a bottom-up approach to mechanically extrapolate human pharmacokinetics from *in vitro* and silicon data.

### 3.3 Physiologically Based Pharmacokinetic Model Validation

There are no clear requirements or guidelines on how to determine the quality of the PBPK model currently. In regulatory guidelines, the criteria for evaluating the effectiveness of a model are usually put forward in the context of whether the model meets the performance requirements for its specific purpose.

**FIGURE 6:**
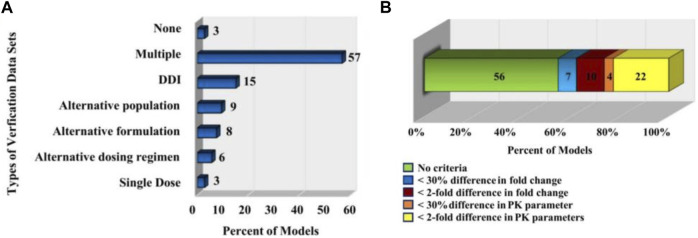
PBPK model validation. **(A)** The types of *in vivo* datasets used to verify the quality of the models (Sager et al., 2015). **(B)** The distribution of the acceptance criteria used in PBPK models (Sager et al., 2015).

As shown in Figure 6A, a statistic (Sager et al., 2015) pointed out that 97% of PBPK related articles between 2008 and 2015 will undergo model verification, but only 32% of the data set used for verification (the data set used for validation here generally describes a pharmacokinetic process) were reported to match the simulated age, gender of the models. In addition, only 21% of model validations took into account genotype. Since some PBPK model parameters are related to characteristics such as age, gender, genotype, etc., this may lead to a decrease in the accuracy of the model prediction and the failure of the model verification.

Statistics (Sager et al., 2015) also pointed out that the criteria for determining model performance of PBPK model in related papers published in English from 2008 to 2015 are inconsistent and subjective. As shown in Figure 6B, in 56% of the models, the authors did not specify a criterion to determine whether their model was successful; for 7% models, the authors specified that the predicted mean PK parameters were supposed to be within 30% of the observed mean; for 10% of the models, the predicted fold change in the AUC or Cmax between different simulated populations or study conditions had to be within two-fold of the observed fold change; for 4% of the models, the predicted fold change in ADME characteristics (e.g., AUC, Cmax.) in a given population were supposed to be within 30% of the observed fold change; for 22% of the models, the predicted ADME characteristics (e.g., AUC, Cmax.) in a given population were supposed to be within two-fold of the observed value.

### 3.4 Application of Physiologically Based Pharmacokinetic Model Combined With Organ-on-a-Chip

#### 3.4.1 Models of Drug Pharmacokinetics in Micro-Physiological System

Conventional flat cell culture, due to its simple and limited structure, is often unable to accurately study the ADME process in the human body. Micro-physiological systems can be used to simulate drug transport and metabolism between human organs. The current MPS calculation model consists of only a few number of organs, usually gut and liver, and other blood supplying organs are represented by mixing pools (Przekwas and Somayaji, 2020).

**FIGURE 7:**
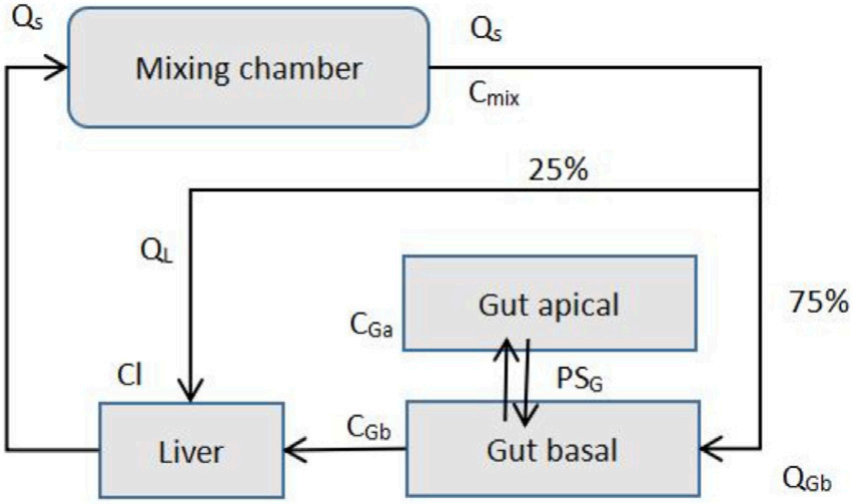
MIT gut-liver MPS Platform (Adapted from Kasendra et al., 2018).

The PK transport model of the MPS platform can be described by ordinary differential equations just like the simple one-compartment model (Jones and Rowland-Yeo, 2013). Only a few ordinary differential equations are used to describe it. Figure 7 presents the description of the transport model of MIT’s gut-liver MPS platform (Kasendra et al., 2018).

Species mass balance equations in each organ: Gut apical (lumen-enterocytes):

$$V_{Ga}\frac{dC_{Ga}}{dt}=P_{G}A_{G}\left(C_{Gb}-C_{Ga}\right)-CL_{G}C_{Ga}$$

Gut basolateral (blood):

$$V_{Ga}\frac{dC_{Gb}}{dt}=Q_{Gb}\left(C_{mix}-C_{Gb}\right)A_{G}\left(G_{Gb}-C_{Ga}\right)$$

Liver:

$$V_{L}\frac{dC_{L}}{dt}=Q_{Gb}\left(G_{Gb}+C_{L}\right)+Q_{L}\left(G_{mix}-C_{L}\right)-CL_{L}C_{L}$$

Mixer:

$$V_{mix}\frac{dC_{mix}}{dt}=\left(Q_{Gb}+G_{L}\right)\left(C_{L}-C_{mix}\right)$$

C_Ga_, C_Gb_, C_L_, and C_mix_ are drug concentrations in individual organs, and V_Ga_, V_Gb_, V_L_, and V_mix_ are the volumes of each organs. AG is the gut barrier exchange area, PG is the gut barrier effective permeability, and CL_G_ and CL_L_ are intrinsic drug clearance rates for the gut and liver. Q is the flow rates, which is determined by the total outflow from the mixer.

#### 3.4.2 *In vitro* to *In vivo* Extrapolation Process

The purpose of building the PBPK model based on the OOC device is to convert drug data from *in vitro* experiments to organ-level data, which is the IVIVE process. Existing method requires us to know the scaling factor when estimating the organ-level data using *in vitro* PK results from static cell culture experiments. Scaling factor is the ratio from vitro experiments data to the organ-level data. But this method has defects such as: 1) Absence of *in vivo* aspects of fluid flow and mechanical cues. 2) No multiscale architecture at the biological interfaces, which is essential to emulating certain organ-level functions. The OoC or HoC devices is an ideal alternative.

Some researchers mentioned some future directions: according to the specific drug properties for a given HoC configuration and design to get the scaling factors, such as the vitro system, medium type, cell properties, cell culture conditions, and drug properties (Prantil-Baun et al., 2018).

##### 3.4.3 Drug-Drug Interactions

**FIGURE 8:**
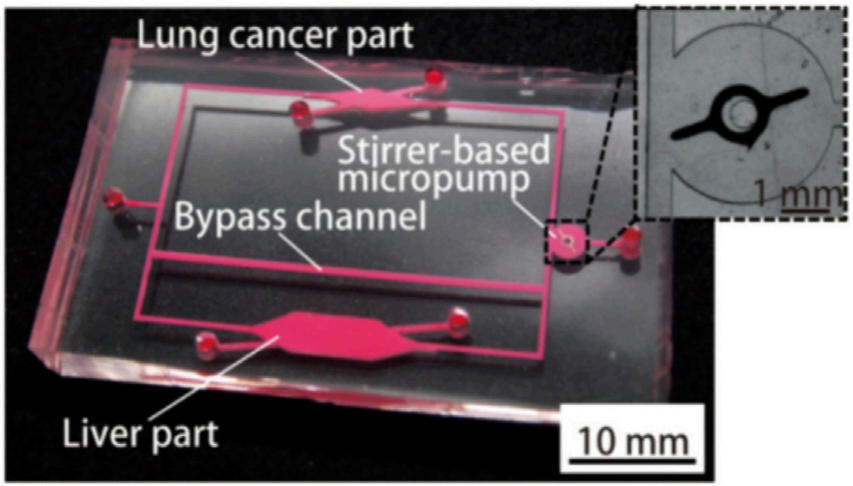
Photograph of the fabricated organs-on-chips with a liver part, a lung cancer part, and other parts connected by micro-channels (Shinha et al., 2020).

Organs-on-a-chip technology has been used in the construction of PBPK models in drug development. A team from Tokai University in Japan introduced a study using organs-on-a-chip technology and PKPD models to evaluate drug-drug interactions (DDI) in 2019 (Shinha et al., 2020) as shown in Figure 8.

Shinha et al. (2020) established a PK-PD model to predict the efficacy of the drug. The change in metabolite concentration can be expressed as:

$$C_{M}\left[t\right]=\frac{E_{p}\times f_{m}\times X_{\circ }}{E_{p}-E_{M}}\times \left(e^{-\frac{Q\times E_{M_{t}}}{V_{d}}\times }-e^{-\frac{Q\times E_{P_{t}}}{V_{d}}\times }\right)$$

The drug efficacy is expressed as:

$$\frac{Celldensity}{Controlcelldensity}=-0.086\times \ln\left(\int_{t}^{0}CM\left[t\right]\right)+0.052$$

where: CL: the clearance in the liver part, *V*_*d*_: the distribution volume, Q: the flow rate in the liver part, E: the extraction ratio in the liver part, fm: the fraction metabolized (he metabolized rate by each metabolic enzyme). The numerical parameters, *V*_*d*_, Q, and X0 were determined from the MOoC design and the experimental conditions.

The cells were exposed to CPT-11 dissolved in the culture medium, which was exchanged every 24 h. After exposure for 72 h, the cells were stained and observed using camera installed with a fluorescence microscope. The extraction ratios in the liver part of CPT-11(Irinotecan Hydrochloride Trihydrate) and SN-38 (7-Ethyl-10-hydroxycamptothecin) in the multiple-organs-on-chips (MOoCs) were estimated from the experimental results using the MOoCs with and without bypass channels and our PK-PD model.

For the evaluation of the DDI, they used CPT-11 and simvastatin (SV) or Ritonavir (RTV) for MOoC at the same time, and observed the changes cell densities on the OoC. The effects of DDI were evaluated by comparing the predicted cell density of the PK-PD model with that of the experimental results.

After analyzing the experimental results and the predicted value of the PKPD model, the researchers found that the DDI of inhibitor chemicals were quite similar to the results of previous studies, they used OoC to evaluate the effect of metabolites from experimental results, and estimate drug-specific parameters by combining PKPD and OoC. Their final conclusion is that the DDI evaluation using the PKPD model and OoC is useful.

##### 3.4.4 Recent Advances in Physiologically Based Pharmacokinetic Modeling Enabled by Organs-on-Chips

As previously mentioned, PDMS is currently the mainstream material used in the fabrication of OoCs. However, its absorption of hydrophobic small molecules remains as a problem and should be resolved before PDMS-made OoC can be put into the practice of drug experiment. Ingber (Grant et al., 2021) proposed a method that combines experiment and simulation to predict the concentration of drugs in OoCs made of PDMS materials.

**FIGURE 9:**
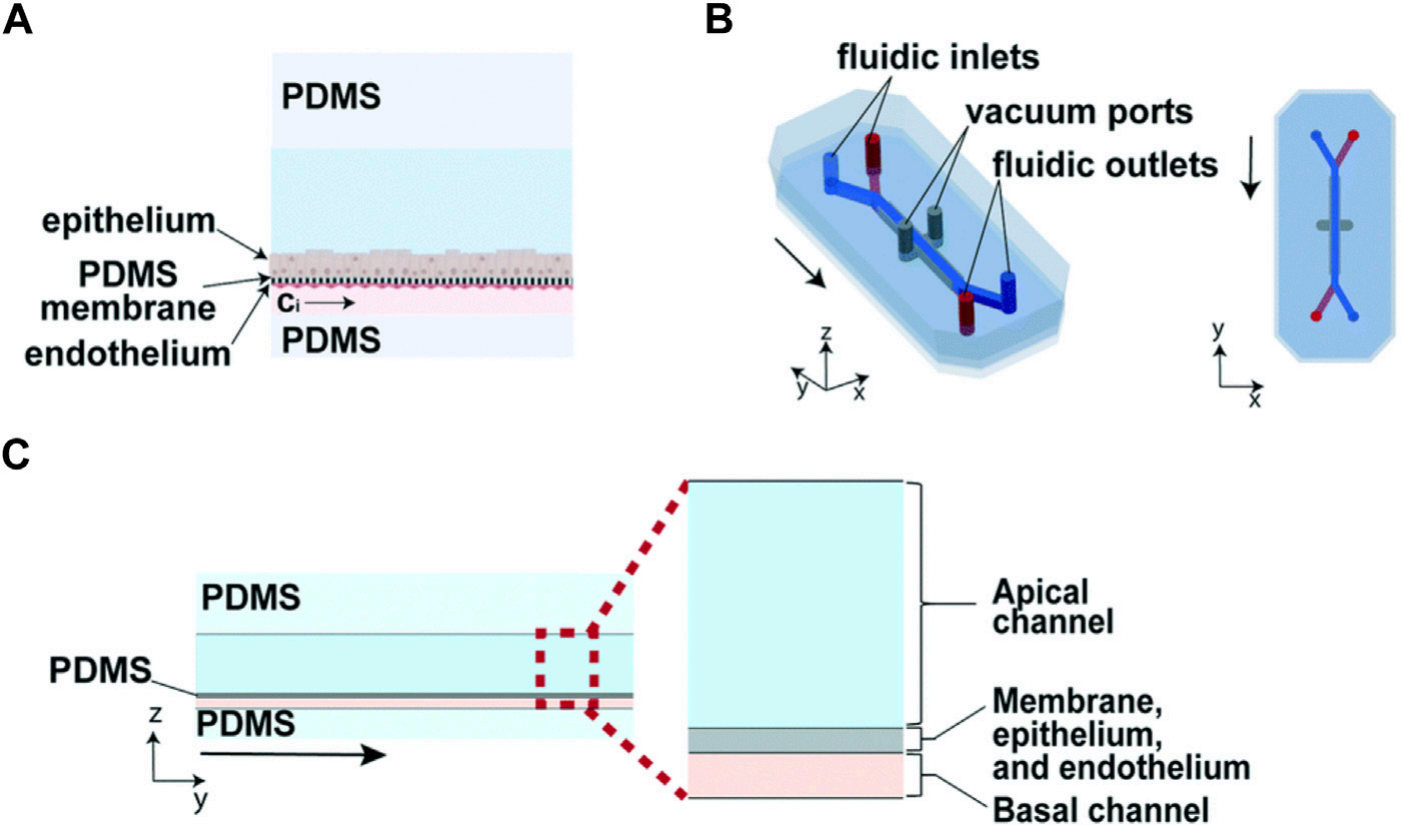
Design of a two-channel human lung airway organ chip. **(A)** reconstruction of the porous PDMS film; **(B)** the illustration of the lung airway organ chip; **(C)** the side view of the construction of organ chip (Grant et al., 2021).

As shown in Figure 9, the organ chip used is a commercially available PDMS microfluidic culture device, which contains two parallel fluid channels separated by a porous PDMS film.

It is assumed that the drug must be first adsorbed to the wall before it enters into the bulk PDMS. The process of the absorption of a drug from the cell culture medium onto the PDMS wall can be described by the partition coefficient (i.e., P):

$$P=\frac{C_{pdms}}{C_{med}}$$

Where C_pdms_ is the drug concentration in PDMS and C_med_ is the drug concentration in cell culture medium. After the drug is adsorbed to the PDMS wall, it diffuses into the bulk PDMS according to Fick’s second law:

$$\rho \frac{\partial c_{med}}{\partial t}-\nabla \left(D_{med}\nabla c_{med}\right)+u\nabla c_{med}=0$$

$$\rho \frac{\partial c_{pdms}}{\partial t}-\nabla \left(D_{pdms}\nabla c_{pdms}\right)=0$$

Where D_med_ is the diffusivity of drug in cell culture medium, D_pdms_ is the diffusivity of drug in PDMS.Since D_pdms_ is not easy to measure, an experimental method for the measure of D_pdms_ is proposed.

The drug intended to investigate is amodiaquine, and fluorescein isothiocyanate (FITC) was used as a surrogate compound to amodiaquine because it is similar in molecular weight, structure, and hydrophobicity. In the designed experiment, FITC is absorbed into the bulk PDMS, and fluorescence microscope can be used for quantitative and spatial analysis. The estimated value of D_pdms_ can be obtained by fitting the analytical solution of Fick second law with experimental data. After obtaining the estimated value of D_pdms_, the estimated value of P can be obtained by fitting experimental data and simulation results. All the parameters needed in the simulation are obtained.

**FIGURE 10:**
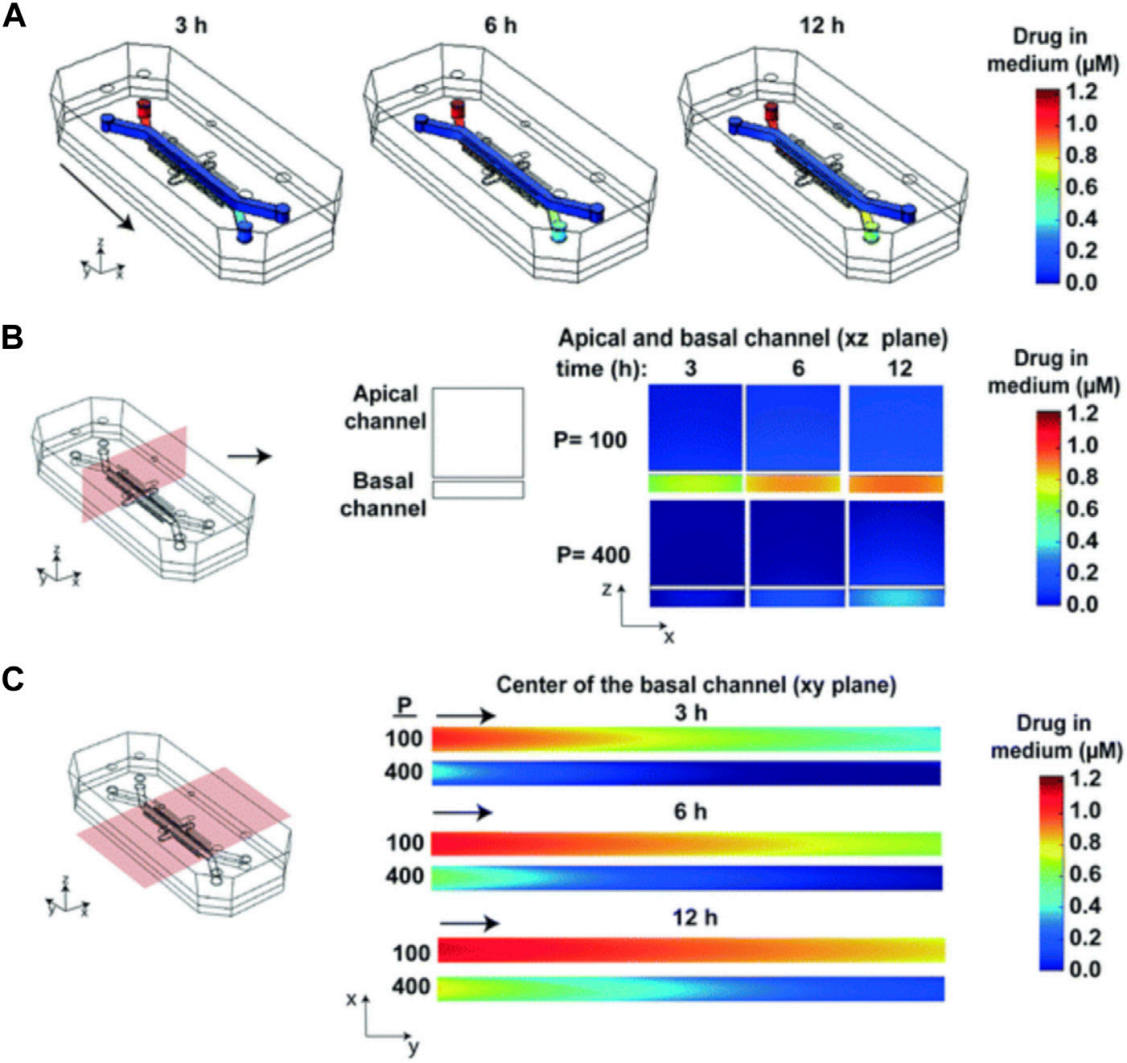
Heat maps of the drug concentration. **(A)** 3D surface heat maps of the concentration of drug in the fluidic channels of the human lung airway chip at 3, 6, and 12 h; **(B)** 2D heat maps of drug concentration through a vertical cross section in the center of the chip; **(C)** 2D heat maps of drug concentration through a horizontal cross section in the center of the basal channel (Grant et al., 2021).

As is shown in Figure 10, subsequent simulations revealed the influence of factors such as drug delivery speed on drug concentration, and a more realistic drug concentration curve was obtained. In short, this article describes a strategy that combines experiment and computational method to simulate the spatial and temporal gradients of drugs without obtaining the distribution coefficient of drugs in PDMS in advance.

## 4 Discussion and Future Perspectives

Physiologically based pharmacokinetic (PBPK) model constructs the mathematical models to study pharmacokinetic (PK) behaviors following compartmental modeling approach with relevant human physiology considered. It provided a great way to predict the ADME (absorption, distribution, metabolism and excretion) process of drugs. Organ-on-a-chip, as an emerging technology aimed to simulate the organ structure and function *in vitro*. The combination of the organ-on-a-chip technology and PBPK PD model provides a new tool in new drug development, personalized medicine and other fields.

### 4.1 Considerable Progress of Physiologically Based Pharmacokinetic Modeling on Organ-on-a-Chips

The application of PBPK model in OoC is still an emerging research area while a few works that combine PK model with OoC for drug development have been reported. In 2017, Tsamandouras et al. first introduced a MOoCs system including gut-on-a-chip and liver-on–a-chip described by the PK model, and used this system to study the mechanism of drug transport (Tsamandouras et al., 2017). *In vitro* to *in vivo* extrapolation (IVIVE) of these results are also provided. This work reveals the bright prospects of the combination of the PK model with OoC in drug development. Shinha et al. used the MOoCs system described by the PBPK model to study the inhibitory effect of simvastatin and ritonavir on the metabolism of CPT-11, thereby demonstrating that the combination of PK model and OoC can be used for drug-drug interaction (DDI) prediction (Shinha et al., 2020). Works including the above studies demonstrate the potentially great application prospects of PBPK modeling on OoC.

The ultimate goal of *in vitro* PBPK/PKPD research with OoC is to establish complementary experimental and computational tools that can translate *in vitro* data to the *in vivo* situation (Przekwas and Somayaji, 2020). However, the differences between abstraction and real situation make it difficult to mimic *in vivo* physiological functions and pharmacological responses (Wikswo et al., 2013a). As two key parts of *in vitro* PK-PD modeling, much efforts should be made on improving the OoC technologies and computational models to move forward, and considerable progress has been achieved.

### 4.2 Current Limitations and Future Improvements

On the OoC side, firstly, due to availability of data on allometric ratios and its simplicity in terms of implementation, allometric scaling has been the most commonly used method. But some researchers have found it difficult in sustaining the allometric ratios upon scale-down (Wikswo et al., 2013b). One example is that some drugs first metabolized by the liver to their active compounds. Under this circumstance, the other organs could not be exposed to realistic levels unless the liver-on-a-chip could provide a physiological conversion of the drug. Likewise, in order to make paracrine signaling between organs replicable, the cytokines produced by each organ should be present in the blood at physiological concentrations. An alternative is scaling organ-on-chips considering the amount of time that each organ is exposed to a molecule (a.k.a. residence time) (van Midwoud et al., 2010). Considering the extent of reaction in the tissue, residence time-based scaling which is based on the degree of chemical conversion has a great advantage over allometric scaling and may better replicate organ–organ interactions.

Secondly, the current organ-on-a-chip platforms are predominately made of by polydimethylsiloxane (PDMS), which has been proved to be attributable for adsorption of biomolecules (Abaci and Shuler, 2015). With growing effort to address this problem, there are now several encouraging solutions available, including modification of the surface chemistry of PDMS to prevent adsorption, using alternative materials (e.g., polystyrene) to PDMS or adjust the computational model for compensation (Prantil-Baun et al., 2018).

On the computational model side, considering the dynamic process of ADME, microfluidic OoC devices require spatially distributed models that account for convection, diffusion, barrier transport, and reaction phenomena, while the conventional *in vitro* static cell cultures could be represented by simple one compartment well-stirred mathematical models (Przekwas and Somayaji, 2020). So in an attempt to achieve more precise simulation, novel mathematical formulation and multiscale models are necessary.

Owning to the complicated nature of the physiological system, current mathematical models of micro- physiological systems (MPS) use the simplified ordinary differential equation based models. Other than individual OoC models, mathematical modeling of MOoC also involves models of various peripheral devices such as tubing, mixers, and reservoirs. Similar to PBPK, simulation of a multi-organ closed-loop MPS involves the “arterial” medium distribution to “venous” medium, and collection from individual organs (Przekwas and Somayaji, 2020). Furthermore, the reservoir model should account for total medium volume dynamics, sampling, fresh medium resupply, and the *in vitro* “intravenous” drug administration. In contrast, current MPS combine both medium pools into a single “mixer” (Przekwas and Somayaji, 2020).

PBPK modeling on OoC has shown great application prospects and is expected to revolutionize the field of drug development and personalized medicine. In China, some start-ups are trying to combine PBPK modeling with OoC to reduce the time cost and opportunity cost of new drug development, and have achieved initial success.

PBPK modeling on OoC provided great experimental and computational tools to understand the ADME process of drugs *in vitro*. There is no doubt that relevant studies would make significant advance in our understanding therapeutic effects of drugs on human pathophysiology. Considerable progress has been made in the field of preclinical trial and personalized medicine based on PBPK/PD modeling and OoC technology. However, much are needed to be done to guarantee the IVIVE process of the fruitful results. Then PBPK modeling on OoC would become an essential part of new drug development.

## Abbreviations

PBPK, physiologically based pharmacokinetic; PD, pharmacodynamics; OoC, Organ-on-a-Chip; MOoCs, Multiple Organs-on-Chips; IVIVE, *in vitro* to *in vivo* extrapolation; IVIVR, *in vitro in vivo* relationship; DDI, Drug-Drug Interaction; ADME, absorption, distribution, metabolism, excretion; PDMS, polydimethylsil.
